# Laparoscopic Cholecystectomy for Severe Acute Cholecystitis in a Patient with Situs Inversus Totalis and Posterior Cystic Artery

**DOI:** 10.1155/2008/465272

**Published:** 2008-05-07

**Authors:** Theodoros E. Pavlidis, Kyriakos Psarras, Apostolos Triantafyllou, Georgios N. Marakis, Athanasios K. Sakantamis

**Affiliations:** Second Propedeutical Department of Surgery, School of Medicine, Aristotle University of Thessaloniki, Constantinoupoleos 49, 546 42 Thessaloniki, Greece

## Abstract

*Situs inversus totalis* is an inherited condition characterized by a mirror-image transposition of thoracic and abdominal organs. It often coexists with other anatomical variations. Transposition of the organs imposes special demands on the diagnostic and surgical skills of the surgeon. We report a case of a 34-year-old female patient presented with left upper quadrant pain, signs of acute abdomen, and unknown *situs inversus totalis*. Severe acute cholecystitis was diagnosed, and an uneventful laparoscopic cholecystectomy was performed. A posterior cystic artery was identified and ligated. Laparoscopic cholecystectomy is feasible in patients with severe acute calculus cholecystitis and *situs inversus totalis*; however, the surgeon should be alert of possible anatomic variations.

## 1. INTRODUCTION

*Situs viscerum inversus totalis* or total transposition of thoracic and abdominal organs is a rare congenital disorder, inherited by an autosomal recessive gene. It appears with an incidence ranging between 1 : 5000 and 1 : 20000 according to the region [1]. It may pose several technical difficulties during operative procedures, especially during laparoscopic operations. The mirror image of laparoscopic view creates unfamiliarity for the surgeon and his usual maneuvers. Additionally, all instrument design is for right-handed surgeons. More than twenty cases of laparoscopic cholecystectomy in patients with *situs inversus totalis* have been reported up to now including a few other laparoscopic procedures, that is, common bile duct exploration, Nissen fundoplication, and appendectomy [1–5].

Severe acute cholecystitis already requires caution and meticulous maneuvers during laparoscopic cholecystectomy, because the anatomy in Callot’s triangle is rather obscure due to inflammation, fibrosis, and adhesions. The risk of injury, mainly of the common bile duct, is increased. If *situs inversus totalis* coexists, the technical difficulties are exacerbated. Furthermore, the increased possibility of other anatomic variations [2] makes the operation further unfamiliar and increases the risk of complications.

We report a case of laparoscopic cholecystectomy for severe acute calculus cholecystitis, in a patient with *situs inversus totalis* and posterior cystic artery, highlighting the condition and the operating difficulties.

## 2. CASE REPORT

A 34-year-old woman was admitted in emergency with left upper quadrant pain which started suddenly three days ago after a heavy meal, fever (38.5°C), nausea, and fatigue. Anamnesis was free of any past illnesses. Clinically, abdominal tenderness, guarding, and rebound tenderness were found in the left upper quadrant; the heart sounds were normal, but audible on the opposite side of the chest. Apart of leucocytosis (19.500/mm^3^), all other laboratory tests were normal. The preoperative electrocardiogram and chest X-rays showed signs of dextrocardia. On ultrasound, the right lobe of the liver and gallbladder were found on the left side; the spleen was visualized on the right. The gallbladder was seen distended, with signs of inflammation, containing several gallstones. Common bile duct was of normal diameter. A CT scan confirmed the diagnosis of acute cholecystitis and *situs inversus totalis*.

**Figure 1:**
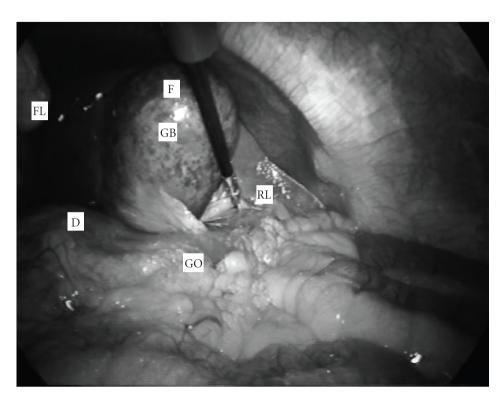
Laparoscopic view of the distended, severely inflamed gallbladder (GB) before evacuative paracentesis. F: fundus, FL: falciform ligament, D: duodenum, RL: right liver lobe, GO: greater omentum.

**Figure 2:**
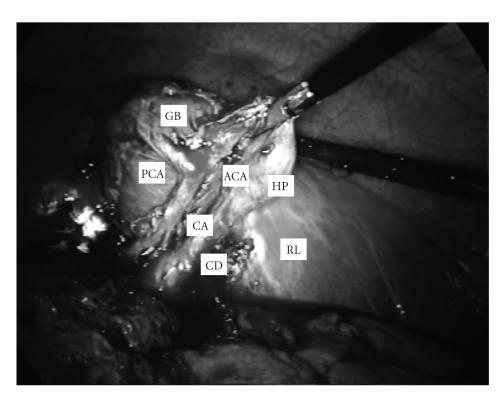
Laparoscopic view of Callot’s triangle after meticulous dissection showing the cystic duct (CD), the cystic artery (CA), the posterior cystic artery (PCA) running to the inferior surface of the gallbladder (GB), and the anterior cystic artery (ACA) running to the Hartman’s pouch (HP). RL: right liver lobe.

Laparoscopic cholecystectomy was performed with the 4-trocar technique, according to the “American” variable. The theatre equipment and the surgical team were positioned reverse. The first trocar (10 mm) with a balloon was inserted under direct vision using the open technique (Hasson’s method) in the subumbilical region. The working trocar (10 mm) was inserted in the usual subxiphoid region and directed to the left of the round ligament; the two grasping trocars (5 mm) were inserted in the usual way but on the left side (middle subcostal and lateral). Pneumoperitoneum was maintained stable at 12 mm Hg. An initial laparoscopy inspected the reversed position of all intraperitoneal viscera. The gallbladder was totally covered by great omentum. After freeing the attachments, the fundus and the upper half of the gallbladder appeared distended with signs of severe inflammation (see Figure 1). Grasping was not possible, therefore an evacuative paracentesis was necessary. Hard adhesions were obscuring the anatomy in Callot’s triangle. Meticulous dissection ensured complete freeing and definition of the course of both cystic duct and cystic artery (see Figure 2). The former was on the left. The latter was on the right, bifurcating into two branches: an anterior cystic branch running to the cystic duct and Hartmann’s pouch and a posterior cystic branch running to the inferior surface of the gallbladder. The gallbladder was partially intrahepatic; its dissection from the liver bed was difficult but uneventful. A drain was placed for 24 hours. The procedure lasted 90 minutes. The gallbladder contained four large stones 2 cm in diameter. Intravenous antibiotic treatment was administered for 48 hours. The patient had an uneventful postoperative course and was discharged on the third day in a nicecondition.

## 3. DISCUSSION

*Situs inversus totalis* may affect the intra-abdominal viscera as well as the intrathoracic organs [1]. It may be associated with many other anatomical variations, more often heart malformations, Kartagener’s syndrome (coexistence with bronchiectasis and sinusitis), liver lobe hypoplasia, biliary atresia in infants, vascular anomalies, and others [1, 2, 6–9]. In a patient with left upper quadrant symptoms, this condition should be kept in mind. Physical examination,electrocardiogram, chest X-rays, and simple abdominal imaging (US, CT) can direct and confirm the diagnosis. CT imaging is an easy examination to quickly reveal anyserious malformations preoperatively, even in the case of an emergency operation. If the patient’s situation permits, it would be very useful to perform a magnetic resonance cholangio-pancreatography (MRCP) or endoscopic retrograde cholangio-pancreatography (ERCP) in order to reveal the exact anatomy of the biliary tract [2].

There have been several reports of laparoscopic cholecystectomy for cholelithiasis in *situs inversus totalis* [1–5, 10–12], and a few others for acute cholecystitis [2, 13]. The encountered difficulties have been well highlighted [1, 10–12]. Briefly, theatre equipment and surgical team must bepositioned on the opposite side than usual (mirror image). The surgeon should keep in mind the mirror image of the anatomy, and he should adjust technique and movements. Before clipping, meticulous dissection and complete clearance of cystic duct and cystic artery from other tissues represent an essential step for a safe procedure. An intraoperative cholangiogram could be very helpful in case of obscure biliary and cystic duct anatomy especially when a preoperative investigation of the biliary tree was not performed [14]. Undoubtedly, the condition needs some extra time to be spent for a safe laparoscopic cholecystectomy. The role of the first assistant for appropriate retraction on Hartmann’s pouch or other elements to facilitate the surgeon’s dissecting maneuvers by his right hand is also very important.

Vascular anomalies of the coeliac trunk and liver are very common in individuals with *situs inversus* [8, 9]. The presence of additional cystic artery branches such as a posterior branch as happened in this case, or an inferior branch in another report [2] may cause unwilling hemorrhage and confusion. Where possible the performance of an abdominal angiography could solve the problem as other investigators have also pointed out [2]. However, this is not always feasible, mainly due to the patient’s condition and in such cases meticulous dissection is the only way for a safe procedure.

In conclusion, laparoscopic cholecystectomy for severe acute calculus cholecystitis in a patient with *situs inversus totalis* and posterior cystic artery is feasible and safe, although technically more demanding. Since the cases treated laparoscopically will be increasing in the future, surgeons should be able to recognize reversed anatomical relationships and coexistence of other anomalies.
